# Subtherapeutic Exposure of Ganciclovir in Children Despite Appropriate Dosing: A Short Communication

**DOI:** 10.1097/FTD.0000000000001050

**Published:** 2022-11-09

**Authors:** Sjanene Marfil, Anne-Grete Märtson, Marlous Toren-Wielema, Coretta Leer-Buter, Elisabeth H. Schölvinck, Jan-Willem C. Alffenaar, Daan J. Touw, Marieke G. G. Sturkenboom

**Affiliations:** * Department of Clinical Pharmacy and Pharmacology, University of Groningen, University Medical Center Groningen, Groningen, the Netherlands;; † University of Liverpool, Antimicrobial Pharmacodynamics and Therapeutics, Liverpool, United Kingdom;; ‡ University of Groningen, University Medical Center Groningen, Groningen, Department of Medical Microbiology and Infection Prevention;; § University of Groningen, University Medical Center Groningen, Beatrix Children's Hospital, Department of Pediatric Infectious Diseases, Groningen, the Netherlands;; ¶ Sydney Institute of Infectious Diseases, the University of Sydney, Westmead;; ‖ The University of Sydney, Sydney Pharmacy School, Faculty of Medicine and Health, Camperdown; and; ** Department of Pharmacy, Westmead Hospital, Westmead, Australia.

**Keywords:** ganciclovir, valganciclovir, therapeutic drug monitoring, cytomegalovirus

## Abstract

Therapeutic drug monitoring (TDM) results for ganciclovir in 12 different treatment episodes showed large intraindividual and interindividual variabilities in the trough concentration and area under the 24-hour concentration–time curve (AUC24). Despite adequate valganciclovir dosing, subtherapeutic concentrations were found in 30% of the treatment episodes. A decrease in viral load was observed regardless of subtherapeutic exposure. These findings show the need for target concentration evaluation and assessment of the applicability of ganciclovir TDM in children.

## INTRODUCTION

Ganciclovir and its oral prodrug valganciclovir are used for the prophylaxis and treatment of cytomegalovirus (CMV) infections in children.^1^ Despite their efficacy, these drugs can lead to severe side effects such as myelosuppression. Suboptimal exposure can lead to drug resistance and, thus, therapy failure.^2–4^

Therapeutic drug monitoring (TDM) is a clinical tool used to monitor drug concentrations and optimize dosing.^5^ TDM could help minimize unwanted effects of ganciclovir and improve efficacy, but evidence-based drug concentration targets are lacking.^6^ Data on the TDM of ganciclovir in children are scarce and have demonstrated varied results.^7–9^ Our study aimed to analyze routine ganciclovir TDM practices in pediatric patients with confirmed CMV infection.

## MATERIALS AND METHODS

We conducted a retrospective study on patients aged <18 years who were treated with valganciclovir for CMV infection between November 2017 and December 2020 at the University Medical Center Groningen (UMCG), Groningen, the Netherlands. The Medical Ethics Review Board of UMCG found the study to be in accordance with Dutch laws because of its retrospective nature (METc 2020/196).

Administration of valganciclovir was performed according to the “Dutch Paediatric Formulary”, based on body surface area, at 900 mg/m^2^/d in 2 doses and adjusted for renal function.^10^ The target minimum concentration (C_min_) was defined as 2–4 mg/L, and the target area under the 24-hour concentration–time curve (AUC_24_) was set at 80–120 mg h/L.^11–15^ We compared and evaluated the initial valganciclovir dosing according to the local and national pediatric dosing guidelines.^10^ Serum ganciclovir concentrations were collected during standard treatment and measured using a validated liquid chromatography with the tandem mass spectrometry LC-MS/MS method.^16^

Pharmacokinetic parameters were calculated using the population pharmacokinetic models of Nguyen et al^17^ and MW/Pharm++ (Prague, Czech Republic). The corresponding AUC24 was calculated for each C_min_ value. If a midconcentration was obtained, the model was used to estimate both the C_min_ and AUC24.

The Spearman rank correlation coefficient test was used to correlate pharmacokinetic parameters with dosing. Statistical significance was set at *P* < 0.05. All data were analyzed using IBM SPSS version 23 (Armonk, NY) and R version 4.0.5.

## RESULTS

Twenty-three patients with 40 different treatment episodes of valganciclovir were included in this study. Some patients had recurrent CMV infection throughout the study period. We categorized the groups into TDM and NO TDM. The patient demographics and clinical outcomes are presented in Table 1. Sixty percent of the treatment episodes in both groups (NO TDM = 17 and TDM = 7) received an initial valganciclovir dose in accordance with the dosing guidelines (maximum of 20% deviation, considering renal function).

**TABLE 1.** Baseline Demographics and Pharmacokinetic Results of Included Patients

A. Individual Patient Characteristics	
Patient Characteristics	Value (N = 23)
No. of male participants (%)	7 (30)
No. of patients with multiple treatment episodes	8 (35)
Age, yr	3.7 (1.4–6.4)
Weight, kg	12.1 (9.4–21.0)
Height, cm	85 (78–116)
No. of episodes per patient	1 (1–2)
Background immunodeficiency, number of occasions with (%)^a^	
Congenital CMV infection	2 (9)
Transplant	
Allogenic stem cell	1 (4)
Lung	2 (9)
Liver	18 (78)

B. Characteristics of Patients Per Treatment Episode				
Variable	No TDM (N = 28)	TDM (N = 12)	*P*	Total (N = 40)
Length of hospital stay, d	0 (0–1)	5 (1–41)	0.002	0 (0–110)
Dose valganciclovir, mg/m^2^/d	811 (652–958)*	720 (677–954)†	0.78	805 (673–961)‡
Duration of therapy, d	42 (28–81)	71 (42–86)	0.17	53 (30–82)
Time to negative viral load, d	20 (13–33)	14 (4–26)†	0.28	20 (11–33)§
C_min_, mg/L		0.6 (0.3–1.6)		
AUC_24_, mg h/L		65 (47–96)		
eGFR, mL/min/1.73 m^2^	114 (75–129)	6 (61–121)	0.07	107 (69–121)

Data are presented as median (IQR) unless denoted as frequency (%)^a^.

N = number of episodes,

* N = 27.

† N = 11.

‡ N = 38.

§ N = 39.

AUC_24_, 24-hour area under the concentration–time curve; C_min_, trough concentration; CMV, cytomegalovirus; eGFR, estimated glomerular filtration rate using the Schwartz formula.

In the TDM group, 30 ganciclovir samples were collected during 12 different CMV episodes (30%). Seventy percent (21 samples) of ganciclovir C_min_ was subtherapeutic (<2 mg/L). The observed median C_min_ (IQR) was 0.6 (0.3–1.6) mg/L, and the median AUC_24_ (IQR) was 65 (47–96) mg h/L.

**FIGURE 1.**
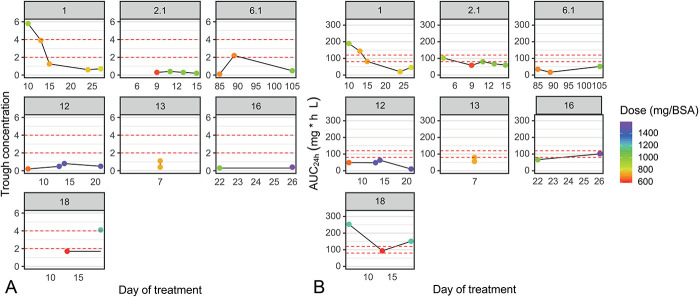
Individual line plots show intraindividual and interindividual variability in C_min_ (A) and AUC_24_ (B); x-axis represents the day of sampling and y-axis (A) C_min_ (mg/L); (B) AUC_24_ (mg h/L); BSA: Body surface area (m^2^); colors indicate valganciclovir dose administered 24 hours before sampling. Red dashed lines represent lower and upper target concentrations (A) or AUC_24_ (B).

No correlation was observed between dosing and C_min_ (*P* = 0.12) or AUC_24_ (*P* = 0.36). Four of the ganciclovir concentrations were in the midrange. These concentrations were extrapolated using Bayesian simulations to estimate the C_min_. Figure 1 shows individual graphs of ganciclovir C_min_ and AUC_24_ of patients who had more than one ganciclovir level measured. These patients showed large intraindividual and interindividual variabilities in dosing and exposure. Moreover, the time of first C_min_ collection in relation to the day of therapy had large variability, with a median of 7 days (IQR 5–13 days).

## DISCUSSION

Overall, our findings suggest that, despite dosing according to the guidelines, pediatric patients are unlikely to achieve optimal drug exposure.^7,9^ However, the optimal drug exposure has not yet been defined for ganciclovir in adults or children. When compared with adults, Luck et al found that children tended to achieve lower ganciclovir C_min_ than adults, possibly due to better renal function.^6^ In that study, a significant number of C_min_ levels in pediatrics were lower than 0.5 mg/L with a risk of ganciclovir resistance and possible treatment failure. Moreover, the suboptimal drug exposure observed in this study is in line with earlier reports where, at most, 30% of the study subjects achieved targeted drug exposure.^12,18,19^

We observed decreasing viral loads (median 14 days to negative viral load), although most of the simulated AUC_24_ values were lower than the target exposure. Our finding is in line with the findings of Launay et al,^8^ where 8 of 10 subjects achieved undetectable viremia with a median AUC_24_ of 35 mg h/L (range, 21–84). In general, the immune system plays an important role in viral clearance. Thus, in some patients with a recovering immune system, a response can be expected without treatment.^20^ However, this was not investigated in this study.

The limitations of this study were its retrospective nature, a small number of participants, and infrequent monitoring of ganciclovir concentrations and CMV viral load. We also used a dosing approach different from that used in previous studies, which makes it difficult to compare the results.^21,22^ It is possible that the lower concentrations can be explained by the lower dosages used in this study, although an additional study with a different dosing approach is required to confirm this.

## CONCLUSIONS

Our results showed large intravariability and intervariability in pharmacokinetics. As most patients receive the standard recommended dose, TDM can be beneficial in reducing the variability in ganciclovir concentrations. Although the lower exposure did not result in treatment failure in our study, this does not imply that the concentrations were optimal. Another small study demonstrated a slow decline in viral load at currently used dosages.^6^ Clearly, there is a need for evidence-based target concentrations for CMV treatment to guide dosing that maximizes viral load reduction without increasing toxicity. Evaluation of an in vitro pharmacokinetic/pharmacodynamic (PK/PD) target in a larger population-based prospective study including frequent PK and virological sampling may aid in exploring the benefits of TDM in children. Moreover, monitoring AUC_24_ is recommended, as it has been reported to be a good indicator of efficacy and toxicity in pediatric patients.^23,24^ This may be beneficial for individual treatment.

This study does not provide a final answer on the value of TDM of ganciclovir, but it raises an important concern about the large intraindividual and interindividual variability in drug exposure, resulting in potential suboptimal treatment in some children.
